# Mortality reduction in older COVID-19-patients hospitalized in Spain during the second pandemic wave from the SEMI-COVID-19 Registry

**DOI:** 10.1038/s41598-023-42735-5

**Published:** 2023-10-18

**Authors:** José-Manuel Casas-Rojo, Juan-Miguel Antón-Santos, Jesús Millán-Núñez-Cortés, Ricardo Gómez-Huelgas, José-Manuel Ramos-Rincón, Manuel Rubio-Rivas, Miguel-Ángel Corrales-González, Maria-Rosa Fernández-Madera-Martínez, José-Luis Beato-Pérez, Francisco Arnalich-Fernández, Cristina Gállego-Lezaun, Pablo Pérez-Martínez, Sonia Molinos-Castro, Yale Tung-Chen, Manuel Madrazo, Manuel Méndez-Bailón, Daniel Monge-Monge, Gema-María García-García, Rosa García-Fenoll, Noemí Gilabert, Rebeca Fuerte-Martínez, Marta Contreras-Sánchez, Nicolás Rhyman, Jorge Peris-García, Carlos Lumbreras-Bermejo, The SEMI-COVID-19 Network, Ana Belén Barbero-Barrera, Blanca Beamonte-Vela, Coralia Bueno-Muiño, Charo Burón-Fernández, Ruth Calderón-Hernáiz, Irene Casado-López, José-Manuel Casas-Rojo, Andrés Cortés-Troncoso, Pilar Cubo-Romano, Francesco Deodati, Alejandro Estrada-Santiago, Gonzalo García-Casasola Sánchez, Elena García-Guijarro, Francisco Javier García Sánchez, Pilar García de la Torre, Mayte de Guzmán García-Monge, Davide Luordo, María Mateos-González, José A. Melero-Bermejo, Cruz Pastor-Valverde, José Luis Pérez-Quero, Fernando Roque-Rojas, Lorea Roteta-García, Elena Sierra-Gonzalo, Francisco Javier Teigell-Muñoz, Juan Vicente de la Sota, Javier Villanueva-Martínez, Laura Abarca Casas, Álvaro Alejandre de Oña, Rubén Alonso Beato, Leyre Alonso Gonzalo, Jaime Alonso Muñoz, Crhistian Mario Amodeo Oblitas, Cristina Ausín García, Marta Bacete Cebrián, Jesús Baltasar Corral, Maria Barrientos Guerrero, Alejandro D. Bendala Estrada, María Calderón Moreno, Paula Carrascosa Fernández, Raquel Carrillo, Sabela Castañeda Pérez, Eva Cervilla Muñoz, Agustín Diego Chacón Moreno, Maria Carmen Cuenca Carvajal, Sergio de Santos, Andrés Enríquez Gómez, Eduardo Fernández Carracedo, María Mercedes Ferreiro-Mazón Jenaro, Francisco Galeano Valle, Alejandra Garcia, Irene Garcia Fernandez-Bravo, María Eugenia García Leoni, María Gómez Antúnez, Candela González San Narciso, Anthony Alexander Gurjian, Lorena Jiménez Ibáñez, Cristina Lavilla Olleros, Cristina Llamazares Mendo, Sara Luis García, Víctor Mato Jimeno, Clara Millán Nohales, Jesús Millán Núñez-Cortés, Sergio Moragón Ledesma, Antonio Muiño Míguez, Cecilia Muñoz Delgado, Lucía Ordieres Ortega, Susana Pardo Sánchez, Alejandro Parra Virto, María Teresa Pérez Sanz, Blanca Pinilla Llorente, Sandra Piqueras Ruiz, Guillermo Soria Fernández-Llamazares, María Toledano Macías, Neera Toledo Samaniego, Ana Torres do Rego, Maria Victoria Villalba Garcia, Gracia Villarreal, María Zurita Etayo, Mª Mar Ayala-Gutiérrez, Rosa Bernal López, José Bueno Fonseca, Verónica Andrea Buonaiuto, Luis Francisco Caballero Martínez, Lidia Cobos Palacios, Clara Costo Muriel, Francis de Windt, Ana Teresa Fernandez-Truchaud Christophel, Paula García Ocaña, Ricardo Gómez Huelgas, Javier Gorospe García, José Antonio Hurtado Oliver, Sergio Jansen-Chaparro, Maria Dolores López-Carmona, Pablo López Quirantes, Almudena López Sampalo, Elizabeth Lorenzo-Hernández, Juan José Mancebo Sevilla, Jesica Martín Carmona, Luis Miguel Pérez-Belmonte, Iván Pérez de Pedro, Araceli Pineda-Cantero, Carlos Romero Gómez, Michele Ricci, Jaime Sanz Cánovas, José-Manuel Ramos-Rincón, Xavier Corbella, Francesc Formiga Pérez, Narcís Homs, Abelardo Montero, Jose María Mora-Luján, Manuel Rubio-Rivas, Victoria Augustín Bandera, Javier García Alegría, Nicolás Jiménez-García, Jairo Luque del Pino, María Dolores Martín Escalante, Francisco Navarro Romero, Victoria Nuñez Rodriguez, Julián Olalla Sierra, Ana María Álvarez Suárez, Carlos Delgado Vergés, Rosa Fernandez-Madera Martínez, Eva Mª Fonseca Aizpuru, Alejandro Gómez Carrasco, Cristina Helguera Amezua, Juan Francisco López Caleya, Diego López Martínez, María del Mar Martínez López, Aleida Martínez Zapico, Carmen Olabuenaga Iscar, Lucía Pérez Casado, María Luisa Taboada Martínez, Lara María Tamargo Chamorro, Jose Luis Beato Pérez, Maria Lourdes Sáez Méndez, Jorge Álvarez Troncoso, Francisco Arnalich Fernández, Francisco Blanco Quintana, Carmen Busca Arenzana, Sergio Carrasco Molina, Aranzazu Castellano Candalija, Germán Daroca Bengoa, Alejandro de Gea Grela, Alicia de Lorenzo Hernández, Alejandro Díez Vidal, Carmen Fernández Capitán, Maria Francisca García Iglesias, Borja González Muñoz, Carmen Rosario Herrero Gil, Juan María Herrero Martínez, Víctor Hontañón, Maria Jesús Jaras Hernández, Carlos Lahoz, Cristina Marcelo Calvo, Juan Carlos Martín Gutiérrez, Monica Martinez Prieto, Elena Martínez Robles, Araceli Menéndez Saldaña, Alberto Moreno Fernández, Jose Maria Mostaza Prieto, Ana Noblejas Mozo, Carlos Manuel Oñoro López, Esmeralda Palmier Peláez, Marina Palomar Pampyn, Maria Angustias Quesada Simón, Juan Carlos Ramos Ramos, Luis Ramos Ruperto, Aquilino Sánchez Purificación, Teresa Sancho Bueso, Raquel Sorriguieta Torre, Clara Itziar Soto Abanedes, Yeray Untoria Tabares, Marta Varas Mayoral, Julia Vásquez Manau, Nicolás Alcalá Rivera, Anxela Crestelo Vieitez, Esther del Corral Beamonte, Jesús Díez Manglano, Isabel Fiteni Mera, Maria del Mar Garcia Andreu, Martin Gericó Aseguinolaza, Cristina Gallego Lezaun, Claudia Josa Laorden, Raul Martínez Murgui, Marta Teresa Matía Sanz, Antonio Pablo Arenas de Larriva, Pilar Calero Espinal, Javier Delgado Lista, Francisco Fuentes-Jiménez, María del Carmen Guerrero Martínez, María Jesús Gómez Vázquez, Jose Jiménez Torres, Laura Limia Pérez, José López-Miranda, Laura Martín Piedra, Marta Millán Orge, Javier Pascual Vinagre, Pablo Pérez-Martinez, María Elena Revelles Vílchez, Angela Rodrigo Martínez, Juan Luis Romero Cabrera, José David Torres-Peña, Maria del Carmen Beceiro Abad, Maria Aurora Freire Romero, Sonia Molinos Castro, Emilio Manuel Paez Guillan, María Pazo Nuñez, Paula Maria Pesqueira Fontan, Ane Andrés Eisenhofer, Ana Arias Milla, Isolina Baños Pérez, Laura Benítez Gutiérrez, Javier Bilbao Garay, Jorge Calderón Parra, Alejandro Callejas Díaz, Erika Camacho Da Silva, MªCruz Carreño Hernández, Raquel Castejón Díaz, María Jesús Citores Sánchez, Carmen Cubero Gozalo, Valentín Cuervas-Mons Martínez, Laura Dorado Doblado, Sara de la Fuente Moral, Alberto Díaz de Santiago, Itziar Diego Yagüe, Ignacio Donate Velasco, Ana María Duca, Pedro Durán del Campo, Gabriela Escudero López, Esther Expósito Palomo, Ana Fernández Cruz, Amy Galán Gómez, Sonia García Prieto, Beatriz García Revilla, Miguel Ángel García Viejo, Javier Gómez Irusta, Patricia González Merino, Edith Vanessa Gutiérrez Abreu, Isabel Gutiérrez Martín, Ángela Gutiérrez Rojas, Andrea Gutiérrez Villanueva, Jesús Herráiz Jiménez, Fátima Ibáñez Estéllez, Pedro Laguna del Estal, Mª Carmen Máinez Sáiz, Carmen de Mendoza Fernández, María Martínez Urbistondo, Fernando Martínez Vera, María Mateos Seirul-lo, Susana Mellor Pita, Patricia A. Mills Sánchez, Esther Montero Hernández, Alberto Mora Vargas, Victor Moreno-Torres Concha, Ignacio Morrás De La Torre, Elena Múñez Rubio, Rosa Muñoz de Benito, Alejandro Muñoz Serrano, Pablo Navarro Palomo, Ilduara Pintos Pascual, Arturo José Ramos Martín-Vegue, Antonio Ramos Martínez, Celia Rodríguez Olleros, Alberto Roldán Montaud, Yolanda Romero Pizarro, Silvia Rosado García, Diana Ruiz de Domingo, David Sánchez Ortiz, Enrique Sánchez Chica, Irene Solano Almena, Elena Suanzes Martin, Yale Tung Chen, Pablo Tutor de Ureta, Ángela Valencia Alijo, Jose Manuel Vázquez Comendador, Juan Antonio Vargas Núñez, Juan Alberto Aguilera Ayllón, Arturo Artero, María del Mar Carmona Martín, María José Fabiá Valls, Maria de Mar Fernández Garcés, Ana Belén Gómez Belda, Ian López Cruz, Manuel Madrazo López, Elisabeth Mateo Sanchis, Jaume Micó Gandia, Laura Piles Roger, Adela Maria Pina Belmonte, Alba Viana García, Inés Armenteros Yeguas, Javier Azaña Gómez, Julia Barrado Cuchillo, Irene Burruezo López, Noemí Cabello Clotet, Alberto E. Calvo Elías, Elpidio Calvo Manuel, Carmen María Cano de Luque, Cynthia Chocron Benbunan, Laura Dans Vilan, Claudia Dorta Hernández, Ester Emilia Dubon Peralta, Vicente Estrada Pérez, Santiago Fernandez-Castelao, Marcos Oliver Fragiel Saavedra, José Luis García Klepzig, Maria del Rosario Iguarán Bermúdez, Esther Jaén Ferrer, Alejandro Maceín Rodríguez, Alejandro Marcelles de Pedro, Rubén Ángel Martín Sánchez, Manuel Méndez Bailón, Sara Miguel Álvarez, Maria José Nuñez Orantos, Carolina Olmos Mata, Eva Orviz García, David Oteo Mata, Cristina Outon González, Juncal Perez-Somarriba, Pablo Pérez Mateos, Maria Esther Ramos Muñoz, Xabier Rivas Regaira, Laura Mª Rodríguez Gallardo, Iñigo Sagastagoitia Fornie, Alejandro Salinas Botrán, Miguel Suárez Robles, Maddalena Elena Urbano, Andrea María Vellisca González, Miguel Villar Martínez, Daniel Monge Monge, Eva María Ferreira Pasos, Alba Varela García, Rafael Aragon Lara, Inmaculada Cimadevilla Fernandez, Juan Carlos Cira García, Gema Maria García García, Julia Gonzalez Granados, Beatriz Guerrero Sánchez, Francisco Javier Monreal Periáñez, Maria Josefa Pascual Perez, Luis Sáez Comet, Laura Letona Giménez, Uxua Asín Samper, Gonzalo Acebes Repiso, José Miguel García Bruñén, Mónica Llorente Barrio, María Aranzazu Caudevilla Martínez, Jesús Javier González Igual, Rosa García Fenoll, María Aguilera García, Ester Alonso Monge, Jesús Álvarez Rodríguez, Claudia Alvarez Varela, Miquel Berniz Gòdia, Marta Briega Molina, Marta Bustamante Vega, Jose Curbelo, Alicia de las Heras Moreno, Ignacio Descalzo Godoy, Alexia Constanza Espiño Alvarez, Ignacio Fernández Martín-Caro, Alejandra Franquet López-Mosteiro, Gonzalo Galvez Marquez, María José García Blanco, Yaiza García del Álamo Hernández, Clara García-Rayo Encina, Noemí Gilabert González, Carolina Guillamo Rodríguez, Nicolás Labrador San Martín, Manuel Molina Báez, Carmen Muñoz Delgado, Pedro Parra Caballero, Javier Pérez Serrano, Laura Rabes Rodríguez, Pablo Rodríguez Cortés, Carlos Rodriguez Franco, Emilia Roy-Vallejo, Monica Rueda Vega, Aresio Sancha Lloret, Beatriz Sánchez Moreno, Marta Sanz Alba, Jorge Serrano Ballesteros, Alba Somovilla, Carmen Suarez Fernández, Macarena Vargas Tirado, Almudena Villa Marti, José Francisco Pascual Pareja, Isabel Perales Fraile, Arturo Muñoz Blanco, Rafael del Castillo Cantero, José Luis Valle López, Isabel Rábago Lorite, Rebeca Fuerte Martínez, Inés Suárez García, Llanos Soler Rangel, Alicia Alonso Álvarez, Olaya Alonso Juarros, Ariadna Arévalo López, Carmen Casariego Castiñeira, Ana Cerezales Calviño, Marta Contreras Sánchez, Ramón Fernández Varela, Santiago J. Freire Castro, Ana Padín Trigo, Rafael Prieto Jarel, Fátima Raad Varea, Ignacio Ramil Freán, Laura Ramos Alonso, Francisco Javier Sanmartín Pensado, David Vieito Porto, Judit Aranda Lobo, Lucía Feria Casanovas, Jose Loureiro Amigo, Miguel Martín Fernández, Isabel Oriol Bermúdez, Melani Pestaña Fernández, Nicolas Rhyman, Nuria Vázquez Piqueras, Marisa Asensio Tomás, David Balaz, David Bonet Tur, Ruth Cañizares Navarro, Paloma Chazarra Pérez, Jesús Corbacho Redondo, Eliana Damonte White, María Escamilla Espínola, Leticia Espinosa Del Barrio, Pedro Jesús Esteve Atiénzar, Carles García Cervera, David Francisco García Núñez, Francisco Garrido Navarro, Vicente Giner Galvañ, Angie Gómez Uranga, Javier Guzmán Martínez, Isidro Hernández Isasi, Lourdes Lajara Villar, Verónica Martínez Sempere, Juan Manuel Núñez Cruz, Sergio Palacios Fernández, Juan Jorge Peris García, Rafael Piñol Pleguezuelos, Andrea Riaño Pérez, José Miguel Seguí Ripoll, Azucena Sempere Mira, Philip Wikman-Jorgensen, Paloma Agudo de Blas, Coral Arévalo Cañas, Blanca Ayuso, José Bascuñana Morejón, Samara Campos Escudero, María Carnevali Frías, Santiago Cossio Tejido, Borja de Miguel Campo, Carmen Díaz Pedroche, Raquel Diaz Simon, Ana García Reyne, Laura Ibarra Veganzones, Lucia Jorge Huerta, Antonio Lalueza Blanco, Jaime Laureiro Gonzalo, Jaime Lora-Tamayo, Carlos Lumbreras Bermejo, Guillermo Maestro de la Calle, Rodrigo Miranda Godoy, Barbara Otero Perpiña, Diana Paredes Ruiz, Marcos Sánchez Fernández, Javier Tejada Montes, Carmen Cortés Saavedra, Jennifer Fernández Gómez, Borja González López, María Soledad Hernández Garrido, Ana Isabel López Amorós, Santiago López Gil, Maria de los Reyes Pascual Pérez, Nuria Ramírez Perea, Andrea Torregrosa García, José Nicolás Alcalá Pedrajas, Antonia Márquez García, Inés Vargas, Irene Arroyo Jiménez, Marina Cazorla González, Marta Cobos-Siles, Luis Corral-Gudino, Pablo Cubero-Morais, María González Fernández, José Pablo Miramontes González, Marina Prieto Dehesa, Pablo Sanz Espinosa, Sonia Casallo Blanco, Jeffrey Oskar Magallanes Gamboa, Cristina Salazar Mosteiro, Andrea Silva Asiain, Miriam García Gómez, Pablo Ramírez Sánchez, Gorka Arroita Gonzalez, Alazne Lartategi Iraurgi, Asier Aranguren Arostegui, Paula Arriola Martínez, Isabel María Portales Fernández, Esther Martinez Becerro, Amalur Iza Jiménez, Cristian Vidal Núñez, María Aparicio López, Eduardo García López, Mª Soledad Azcona Losada, Beatriz Ruiz Estévez, Ana Maria Alguacil Muñoz, Marta Blanco Fernández, Veronica Cano, Ricardo Crespo Moreno, Fernando Cuadra Garcia-Tenorio, Blanca Díaz-Tendero Nájera, Raquel Estévez González, María Paz García Butenegro, Alberto Gato Díez, Verónica Gómez Caverzaschi, Piedad María Gómez Pedraza, Julio González Moraleja, Raúl Hidalgo Carvajal, Patricia Jiménez Aranda, Raquel Labra González, Áxel Legua Caparachini, Pilar Lopez Castañeyra, Agustín Lozano Ancin, Jose Domingo Martin Garcia, Cristina Morata Romero, María Jesús Moya Saiz, Helena Moza Moríñigo, Gemma Muñiz Nicolás, Enriqueta Muñoz Platon, Filomena Oliveri, Elena Ortiz Ortiz, Raúl Perea Rafael, Pilar Redondo Galán, María Antonia Sepulveda Berrocal, Vicente Serrano Romero de Ávila, Pilar Toledano Sierra, Yamilex Urbano Aranda, Jesús Vázquez Clemente, Carmen Yera Bergua, Andrés de la Peña Fernández, Almudena Hernández Milián, María Areses Manrique, Ainara Coduras Erdozain, Ane Labirua-Iturburu Ruiz, Francisco Javier Bejarano Luque, Francisco-Javier Carrasco-Sánchez, Mercedes de-Sousa-Baena, Jaime Díaz Leal, Aurora Espinar Rubio, Maria Franco Huertas, Juan Antonio García Bravo, Andrés Gonzalez Macías, Encarnación Gutiérrez Jiménez, Alicia Hidalgo Jiménez, Constantino Lozano Quintero, Carmen Mancilla Reguera, Francisco Javier Martínez Marcos, Francisco Muñoz Beamud, Maria Pérez-Aguilar, Alícia Pérez Jiménez, Virginia Rodríguez Castaño, Alvaro Sánchez dedel AlcazarRío, Leire Toscano Ruiz, Diana Alegre González, Irene Ariño Pérez de Zabalza, Sergio Arnedo Hernández, Jorge Collado Sáenz, Beatriz Dendariena, Marta Gómez del Mazo, Iratxe Martínez de Narvajas Urra, Sara Martínez Hernández, Estela Menendez Fernández, Jose Luís Peña Somovilla, Elisa Rabadán Pejenaute, Jesús Ballano Rodríguez-Solís, Luis Cabeza Osorio, María del Pilar Fidalgo Montero, Mª Isabel Fuentes Soriano, Erika Esperanza Lozano Rincón, Ana Martín Hermida, Jesús Martínez Carrilero, José Ángel Pestaña Santiago, Manuel Sánchez Robledo, Patricia Sanz Rojas, Nahum Jacobo Torres Yebes, Vanessa Vento, Luis Fernando Abrego Vaca, Ana Andréu Arnanz, Octavio Arce García, Marta Bajo González, Pablo Borque Sanz, Alberto Cozar Llisto, Sonia de Pedro Baena, Beatriz Del Hoyo Cuenda, Martin Fabregate-Fuente, María Alejandra Gamboa Osorio, Isabel García Sánchez, Andrés González García, Oscar Alberto López Cisneros, Luis Manzano, Miguel Martínez-Lacalzada, Borja Merino Ortiz, Jimena Rey-García, Elisa Riera González, Cristina Sánchez Díaz, Grisell Starita Fajardo, Cecilia Suárez Carantoña, Adrian Viteri-Noël, Svetlana Zhilina Zhilina, Gloria María Alonso Claudio, Víctor Barreales Rodríguez, Cristina Carbonell Muñoz, Adela Carpio Pérez, María Victoria Coral Orbes, Daniel Encinas Sánchez, Sandra Inés Revuelta, Miguel Marcos Martín, José Ignacio Martín González, José Ángel Martín Oterino, Leticia Moralejo Alonso, Sonia Peña Balbuena, María Luisa Pérez García, Ana Ramon Prados, Beatriz Rodríguez-Alonso, Ángela Romero Alegría, Maria Sanchez Ledesma, Rosa Juana Tejera Pérez, Julio César Blázquez Encinar, Carmen Martínez Cilleros, Isabel Jiménez Martínez, Teresa García Delange, Raquel Fernández González, Amara Gonzalez Noya, Carlos Hernández Ceron, Isabel Izuzquiza Avanzini, Ana Latorre Diez, Pablo López Mato, Ana María Lorenzo Vizcaya, Daniel Peña Benítez, Milagros María Peña Zemsch, Lucía Pérez Expósito, Marta Pose Bar, Lara Rey González, Laura Rodrigo Lara, Dafne Cabañero, María Calabuig Ballester, Pascual Císcar Fernández, Ricardo Gil Sánchez, Marta Jiménez Escrig, Cristina Marín Amela, Laura Parra Gómez, Carlos Puig Navarro, José Antonio Todolí Parra, Carlota Tuñón de Almeida, María Esther Fraile Villarejo, Victoria Palomar Calvo, Sara Pintos Otero, Beatriz García López, Carlos Aldasoro Frías, Víctor Madrid Romero, Luis Arribas Pérez, Emilia Martínez Velado, Raquel Aranega González, Ramon Boixeda, Javier Fernández Fernández, Carlos Lopera Mármol, Marta Parra Navarro, Ainhoa Rex Guzmán, Aleix Serrallonga Fustier, José López Castro, Manuel Lorenzo López Reboiro, Cristina Sardiña González, Enrique Rodilla Sala, Jose María Pascual Izuel, Zineb Karroud Zamrani, Hortensia Alvarez Diaz, Tamara Dalama Lopez, Estefania Martul Pego, Carmen Mella Pérez, Ana Pazos Ferro, Sabela Sánchez Trigo, Dolores Suarez Sambade, Maria Trigas Ferrin, Maria del Carmen Vázquez Friol, Laura Vilariño Maneiro, Begoña Cortés Rodríguez, María Esther Guisado Espartero, Lorena Montero Rivas, Maria de la Sierra Navas Alcántara, Raimundo Tirado-Miranda, Marta Nataya Solís Marquínez, Víctor Arenas García, Demelsa Blanco Suárez, Natalia García Arenas, Paula Martínez García, David Castrodá Copa, Andrea Álvarez García, Jaime Casal Álvarez, María Jose Menéndez Calderón, Raquel García Noriega, María Caño Rubia, Joaquin Llorente García, Luis Trapiella Martínez, José Ferreiro Celeiro, Diego Eduardo Olivo Aguilar, Irene Maderuelo Riesco, Juan Valdés Bécares, Alba Barragán Mateos, Andrés Astur Treceño García, Joaquín Delgado Casamayor, Diego García Silvera, Andrea Afonso Díaz, Carolina Hernández Carballo, Alicia Tejera, María José Monedero Prieto, María Blanca Monereo Muñoz, José Manuel Del Arco Delgado, Daniel Rodríguez Díaz, Marta Bethencourt Feria, Francisco Javier Herrera Herrera, María de la Luz Padilla Salazar, Rubén Hernández Luis, Eduardo Mauricio Calderón Ledezma, María del Mar López Gámez, Laura Torres Hernández, Sara Castaño Pérez, Selena Gala Aguilera García, Guillermo Castro Gainett, Alba Gómez Hidalgo, Julia Marfil Daza, Marcelino Hayek Peraza, Reyes Aparicio Santos, Máximo Bernabeu-Wittel, Santiago Rodríguez Suárez, María Nieto, Luis Giménez Miranda, Rosa María Gámez Mancera, Fátima Espinosa Torre, Carlos Hernandez Quiles, Concepción Conde Guzmán, Juan Delgado de la Cuesta, Jara Eloisa Ternero Vega, María del Carmen López Ríos, Pablo Díaz Jiménez, Bosco Baron Franco, Carlos Jiménez de Juan, Sonia Gutiérrez Rivero, Julia Lanseros Tenllado, Verónica Alfaro Lara, Aurora González Estrada, Javier Ena, José Enrique Gómez Segado, Ruth Gonzalez Ferrer, Virginia Gracia Lorenzo, Raquel Monsalvo Arroyo, Marcos Guzmán García, Francisco Javier Vicente Hernández, Ángel Luis Martínez González, Beatriz Vicente Montes, Rosario María García Die, Alberto Muela Molinero, Manuel Martín Regidor, Raquel Rodríguez Díez, Bárbara Hernández Sierra, Luis Felipe Díez García, Iris El Attar Acedo, Carmen Mar Sánchez Cano, Virginia Herrero García, Berta Román Bernal, Júlia Calvo Jiménez, Emmanuel Coloma Bazán, Aina Capdevila Reniu, Joan Ribot Grabalosa, Joaquim Fernández Solà, Irene Carbonell De Boulle, Cristina Gabara Xancó, Olga Rodríguez Núñez, Carlos Jorge Ripper, Anyuli Gracia Gutiérrez, Leticia Esther Royo Trallero, Marta Fernández-Ayala Novo, José Javier Napal Lecumberri, Nuria Puente Ruiz, Jose Riancho, Isabel Sampedro García, Pablo Conde Baena, Joaquín Escobar Sevilla, Laura Gallo Padilla, Patricia Gómez Ronquillo, Pablo González Bustos, María Navío Botías, Jessica Ramírez Taboada, Mar Rivero Rodríguez, Víctor Asensi Alvarez, Noelia Morán Suárez, Sara Rodríguez Suárez, Silvia Suárez Díaz, Lucia Suárez Pérez, Maria Folgueras Gómez, Claudia Moran Castaño, Lucía Meijide Rodríguez, Carlos Vázquez, Itxasne Cabezón Estévanez, Carmen Yllera Gutiérrez, Maria Martinez Sela, Sara Fuente Cosío, César Manuel Gallo Álvaro, Julia Lobo García, Antía Pérez Piñeiro, Yolanda Casillas Viera, Lucía Cayuela Rodríguez, Carmen de Juan Alvarez, Gema Flox Benitez, Laura García Escudero, Juan Martin Torres, Patricia Moreira Escriche, Susana Plaza Canteli, M. Carmen Romero Pérez, Jorge Andrés Soler, Marián Bennasar Remolar, Alejandro Cardenal Álvarez, Daniela Díaz Carlotti, María José Esteve Gimeno, Sergio Fabra Juana, Paula García López, María Teresa Guinot Soler, Daniela Palomo de la Sota, Guillem Pascual Castellanos, Ignacio Pérez Catalán, Celia Roig Martí, Paula Rubert Monzó, Javier Ruiz Padilla, Nuria Tornador Gaya, Jorge Usó Blasco, M. Angeles Martinez Pascual, Leyre Jorquer Vidal, Ana Alberich Conesa, Mari Cruz Almendros Rivas, Miquel Hortos Alsina, José Marchena Romero, Anabel Martin-Urda Diez-Canseco, Francisco Amorós Martínez, Erika Ascuña Vásquez, José Carlos Escribano Stablé, Adriana Hernández Belmonte, Ana Maestre Peiró, Raquel Martínez Goñi, M. Carmen Pacheco Castellanos, Bernardino Soldan Belda, David Vicente Navarro, Ana Suárez Lombraña, Jon Cabrejas Ugartondo, Ana Belén Mancebo Plaza, Arturo Noguerado Asensio, Bethania Pérez Alves, Natalia Vicente López, Marta León Téllez, Francisco Epelde, Isabel Torrente, Pablo Guisado Vasco, Ana Roda Santacruz, Ana Valverde Muñoz, Mª José Esteban Giner, Alejo Erice Calvo-Sotelo, Eva García Sardón, Javier Galán González, Luis Gámez Salazar, Angela Agea Garcia, Itziar Montero Días, Alvaro Santaella Gomez, Marta Correa Matos, Selene Núñez Gaspar, Antonio González Nieto, Raquel Gómez Méndez, Ana Rodríguez Álvarez, Onán Pérez Hernández, Alina Pérez Ramírez, María Candelaria Martín González, Miguel Nicolas Navarrete Lorite, Lourdes González Navarrete, Julio Cesar Alvisa Negrin, José Fernando Armas González, Iballa Jiménez, Paula Ortega Toledo, Esther Martin Ponce, Xjoylin Teresita Egües Torres, Sara Gutiérrez González, Cristina Novoa Fernández, Pablo Tellería Gómez, Oriol Alonso Gisbert, Mercé Blázquez Llistosella, Pere Comas Casanova, Angels Garcia Flores, Anna Garcia Hinojo, Ana Inés Méndez Martínez, Maria del Carmen Nogales Nieves, Agnés Rivera Austrui, Alberto Zamora Cervantes, Vanesa Alende Castro, Ana María Baz Lomba, Ruth Brea Aparicio, Marta Fernández Morales, Jesús Manuel Fernández Villar, María Teresa López Monteagudo, Cristina Pérez García, Lorena Rodríguez Ferreira, Diana Sande Llovo, Maria Begoña Valle Feijoo, Juan Antonio Montes Romero, Jose Luis Serrano Carrillo de Albornoz, Manuel Jesus Soriano Pérez, Encarna Sánchez Martín, Thamar Capel Astrua, Paola Tatiana Garcia Giraldo, Maria Jesús González Juárez, Victoria Marquez Fernandez, Ada Viviana Romero Echevarry, José F. Varona Arche, María Gloria Rojano Rivero, Adrián Montaño Martínez, Reina Valle Bernad, Cristina Limia, Cristina Amado Fernández, Andrea Tejero Fernández, Lucia Paz Fajardo, Tomás de Vega Santos, Antonio López Ruiz, Hector Meijide Míguez

**Affiliations:** 1 Internal Medicine Department, Infanta Cristina University Hospital, Parla, Madrid Spain; 2 Instituto de Investigación Sanitaria Puerta de Hierro-Segovia de Arana (IDIPHISA), Universidad Complutense de Madrid, Madrid, Spain; 3 Internal Medicine Department, Gregorio Marañón University Hospital, Madrid, Spain; 4 Internal Medicine Department, Regional University Hospital of Málaga, Biomedical Research Institute of Málaga (IBIMA), University of Málaga (UMA), Málaga, Spain; 5 Clinical Medicine Department, Miguel Hernandez University of Elche, Ctra N332 s/n, 03550 Sant Joan d’Alacant, Alicante Spain; 6 Internal Medicine Department, Bellvitge University Hospital, L’Hospitalet de Llobregat, Barcelona Spain; 7 Internal Medicine Department, Costa del Sol Hospital, Marbella, Málaga Spain; 8 Internal Medicine Department, Cabueñes University Hospital, Gijón, Asturias Spain; 9 Internal Medicine Department, Complejo Hospitalario Universitario de Albacete, Albacete, Spain; 10 Internal Medicine Department, Hospital Universitario La Paz, Madrid, Spain; 11 Internal Medicine Department, Hospital Royo Villanova, Zaragoza, Spain; 12 Lipids and Atherosclerosis Unit, Department of Internal Medicine, Maimonides Biomedical Research Institute of Córdoba (IMIBIC), Reina Sofía University Hospital, University of Córdoba, CIBER Fisiopatología de la Obesidad y Nutrición (CIBEROBN), Instituto de Salud Carlos III (ISCIII), Madrid, Spain; 13 Internal Medicine Department, Complejo Hospitalario Universitario de Santiago, A Coruña, Spain; 14 Internal Medicine Department, Hospital Universitario Puerta de Hierro Majadahonda, Madrid, Spain; 15 Internal Medicine Department, Hospital Universitario Doctor, Peset, Valencia Spain; 16 Internal Medicine Department, Hospital Clínico San Carlos, Madrid, Spain; 17 Internal Medicine Department, Complejo Asistencial de Segovia, Segovia, Spain; 18 Internal Medicine Department, Complejo Hospital Universitario de Badajoz, Badajoz, Spain; 19 Internal Medicine Department. Hospital, Universitario Miguel Servet, Zaragoza, Spain; 20 Internal Medicine Department, Hospital Universitario de la Princesa, Madrid, Spain; 21 Internal Medicine Department, Hospital Universitario Infanta Sofía, SS de los Reyes, Madrid Spain; 22 Internal Medicine Department, Complexo Hospitalario Universitario A Coruña, A Coruña, Spain; 23 Internal Medicine Department, Hospital de Sant Joan Despí Moisès Broggi, Sant Joan Despí, Barcelona Spain; 24 Internal Medicine Department, Hospital Universitari Sant Joan d’Alacant, Alicante, Spain; 25 Internal Medicine Department, 12 de Octubre University Hospital, Madrid, Spain; 26 Hospital General Uuniversitario de Elda, Alicante, Spain; 27 H. de Pozoblanco, Córdoba, Spain; 28 Hospital. U. Río Hortega, Valladolid, Spain; 29 Hospital Nuestra Señora del Prado, Talavera de la Reina, Toledo Spain; 30 H. de Urduliz Alfredo Espinosa, Vizcaya, Spain; 31 H. Virgen de la Salud, Toledo, Spain; 32 H. U. Son Llàtzer, Palma de Mallorca, Spain; 33 H. Santa Marina, Bilbao, Spain; 34 H. Juan Ramón Jiménez, Huelva, Spain; 35 H. San Pedro, Logroño, La Rioja Spain; 36 H. del Henares, Coslada, Madrid Spain; 37 H. U. Ramón y Cajal, Madrid, Spain; 38 C. A. U. de Salamanca, Salamanca, Spain; 39 H. U. Torrevieja, Alicante, Spain; 40 H. HLA Moncloa, Madrid, Spain; 41 C. H. U. Ourense, Ourense, Spain; 42 H. U. La Fe. Valencia, Valencia, Spain; 43 C. Asistencial de Zamora, Zamora, Spain; 44 H. de Mataró, Barcelona, Spain; 45 H. Público de Monforte de Lemos, Lugo, Spain; 46 H. de Sagunto, Valencia, Spain; 47 C. H. U. de Ferrol, A Coruña, Spain; 48 H. Alto Guadalquivir, Andújar, Jaén Spain; 49 H. Infanta Margarita, Cabra, Córdoba Spain; 50 H. U. San Agustin, Avilés, Asturias Spain; 51 H. Univ. Ntra. Sra. Candelaria, Sta. Cruz de Tenerife, Spain; 52 H. U. Virgen del Rocío, Seville, Spain; 53 H. Marina Baixa, Villajoyosa, Alicante Spain; 54 H. del Tajo, Aranjuez, Madrid Spain; 55 H. San Juan de la Cruz, Úbeda, Jaén Spain; 56 C. Asist. Univ. de León, León, Spain; 57 H. Torrecárdenas, Almería, Spain; 58 H. Dr. José Molina Orosa, Lanzarote, Arrecife Spain; 59 H. Clinic Barcelona, Barcelona, Spain; 60 H. Insular de Gran Canaria, Las Palmas G. C., Spain; 61 H. General Defensa, Zaragoza, Spain; 62 H. U. Marqués de Valdecilla, Santander, Spain; 63 H. U. Virgen de las Nieves, Granada, Spain; 64 H. U. C. de Asturias, Oviedo, Spain; 65 H. Valle del Nalón, Riaño-Langreo, Asturias Spain; 66 H. U. Severo Ochoa, Leganés, Madrid Spain; 67 H. G. U. de Castellón, Castelló de La Plana, Spain; 68 H. Francesc de Borja, Gandía, Valencia Spain; 69 H. de Palamós, Gerona, Spain; 70 H. U. del Vinalopó, Elche, Alicante Spain; 71 H. Platón, Barcelona, Spain; 72 H. U. del Sureste, Arganda del Rey, Madrid Spain; 73 H. Santa Bárbara, Soria, Spain; 74 H. Parc Tauli, Sabadell, Barcelona Spain; 75 H. U. Quironsalud Madrid, Madrid, Spain; 76 H. Virgen de los Lirios, Alcoy, Alicante Spain; 77 H. Asepeyo Coslada, Madrid, Spain; 78 H. San Pedro de Alcántara, Cáceres, Spain; 79 H. U. Lucus Augusti, Lugo, Spain; 80 H. U. de Canarias, Sta. Cruz de Tenerife, Spain; 81 H. Clínico Universitario de Valladolid, Valladolid, Spain; 82 H. Comarcal de Blanes, Girona, Spain; 83 H. do Salnes. Vilagarcía de Arousa, Pontevedra, Spain; 84 H. de Poniente, El Ejido, Almería Spain; 85 H. Virgen del Mar, Madrid, Spain; 86 H. U. HM Montepríncipe, Madrid, Spain; 87 H. Infanta Elena, Huelva, Spain; 88 H. de Montilla, Córdoba, Spain; 89 H. Sierrallana, Torrelavega, Cantabria Spain; 90 H. de la Axarquía, Vélez-Málaga, Málaga Spain; 91 H. Quironsalud A Coruña, A Coruña, Spain

**Keywords:** Infectious diseases, Viral infection

## Abstract

In 2020, the COVID-19 pandemic followed a two-wave pattern in most countries. Hospital admission for COVID-19 in one wave or another could have affected mortality, especially among the older persons. The objective of this study was to evaluate whether the admission of older patients during the different waves, before SARS-CoV-2 vaccination was available, was associated with a different mortality. We compared the mortality rates of patients hospitalized during 2020 before (first wave) and after (second wave) July 7, 2020, included in the SEMI-COVID-19 Registry, a large, multicenter, retrospective cohort of patients admitted to 126 Spanish hospitals for COVID-19. A multivariate logistic regression analysis was performed to control for changes in either the patient or disease profile. As of December 26, 2022, 22,494 patients had been included (17,784 from the first wave and 4710 from the second one). Overall mortality was 20.4% in the first wave and 17.2% in the second wave (risk difference (RD) − 3.2%; 95% confidence interval (95% CI) − 4.4 to − 2.0). Only patients aged 70 and older (10,973 patients: 8571 in the first wave and 2386 in the second wave) had a significant reduction in mortality (RD − 7.6%; 95% CI − 9.7 to − 5.5) (unadjusted relative risk reduction: 21.6%). After adjusting for age, comorbidities, variables related to the severity of the disease, and treatment received, admission during the second wave remained a protective factor. In Spain, patients aged 70 years and older admitted during the second wave of the COVID-19 pandemic had a significantly lower risk of mortality, except in severely dependent persons in need of corticosteroid treatment. This effect is independent of patient characteristics, disease severity, or treatment received. This suggests a protective effect of a better standard of care, greater clinical expertise, or a lesser degree of healthcare system overload.

## Introduction

The coronavirus disease 19 (COVID-19) pandemic, caused by severe acute respiratory syndrome coronavirus 2 (SARS-CoV-2), has become a major, life-changing event that continues to overwhelm man’s collective mind. As of June 15, 2021, 175,333,154 infections and 3,793,230 deaths had been reported worldwide^1^. In 2020, most countries experienced at least two waves of the pandemic.

Patients with COVID-19 who require hospitalization have a high mortality rate. The in-hospital mortality rate was very high in the early phases of the pandemic, though there was a great degree of variability among countries and even areas, with mortality rates ranging from 12% up to 28%^2–6^. Mortality is higher in older patients (especially those over 79 years)^5,7^ as well as in patients with certain underlying conditions^8^.

Mortality is one of the main measures of severity of any epidemic. It is prone to change over time due to improved comprehension of the disease and the development of better treatments. COVID-19 mortality has fluctuated over time^9–11^ and varies according to geography^12,13^. In most countries, the pandemic followed a two-wave pattern in 2020, with a first wave in the spring and a second wave starting in late summer or early autumn. In North America^10^ and Europe^14–16^, mortality was higher in the first wave whereas in Africa^17^ and Brazil^18^, it was higher in the second wave.

In Spain, this evolution has yet to be thoroughly studied. The first wave was followed by three more waves (in early autumn, late autumn, and midwinter of 2020), each of which had lower death tolls and unadjusted mortality rates. A single-center study in Reus, Spain^19^ has shown that, after controlling for known mortality factors, there was still a lower mortality rate in the second wave.

According to our preliminary data, we hypothesized that hospital admission in the different waves could affect the mortality of patients with COVID-19, especially the older people. The primary aim of the study was to evaluate whether the admission of older patients during the different waves of 2020 was associated with a different mortality rate and whether this could be explained by differences in the characteristics of either the patients or the severity of the disease.

## Results

### Mortality estimation and case fatality rates in first and second waves

**Figure 1:**
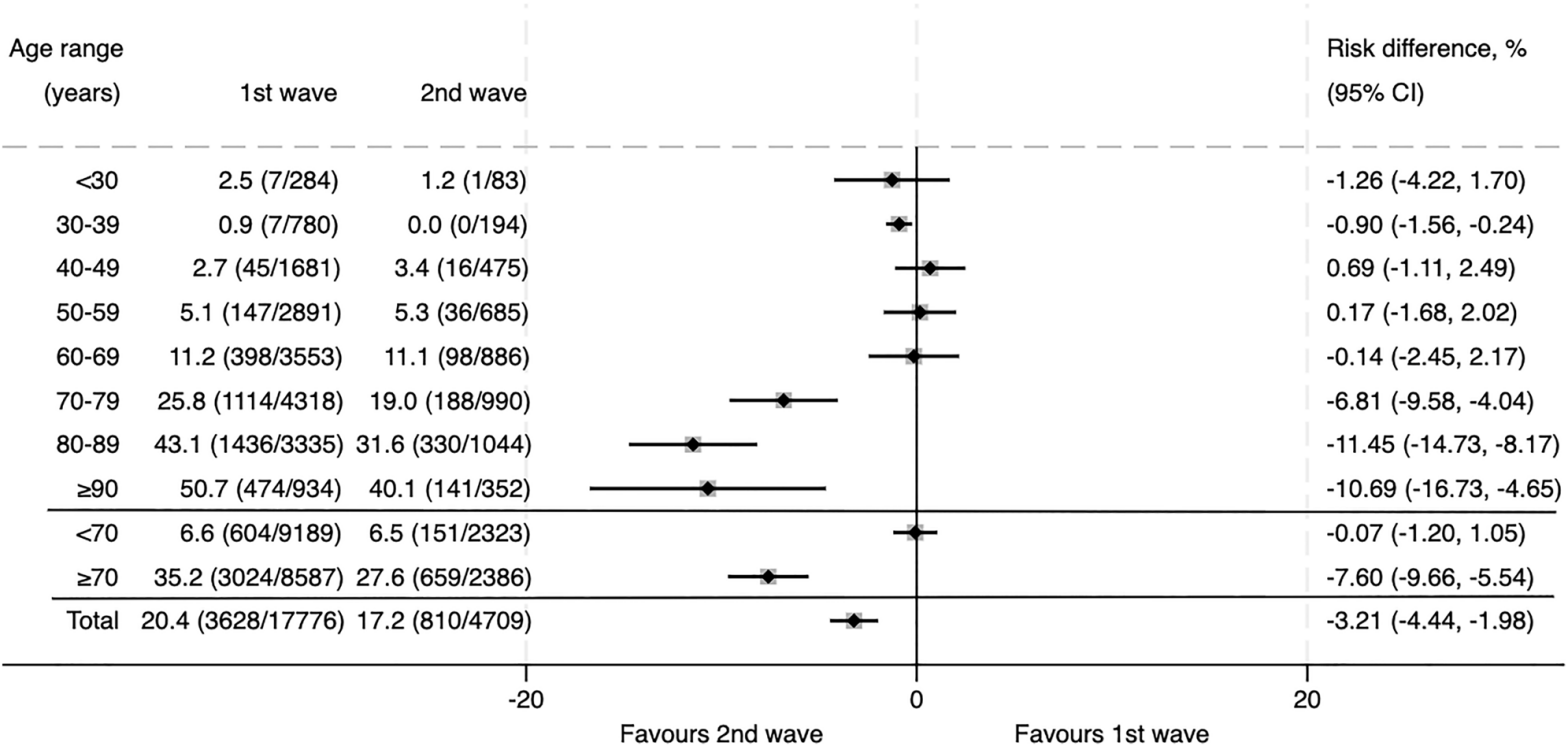
Case Fatality Rate (CFR) according to age in patients hospitalized during the first and second waves of COVID-19, expressed as percentage (deceased patients/total patients). The diamonds indicate the point estimate of the risk difference, and the horizontal bars represent its 95% confidence interval.

As of December 26, 2020, a total of 22,494 patients had been included in the SEMI-COVID-19 Registry: 17,784 patients from the first wave and 4710 patients from the second wave. Overall mortality was 20.4% in the first wave and 17.2% in the second wave (RD − 3.2%; 95% CI − 4.4 to − 2.0). Figure 1 shows the case fatality rate stratified by age. As there were no differences in mortality according to wave in patients younger than 70 years, we focused the study on patients who were 70 years of age and older (10,973 patients: 8587 from the first wave and 2386 from the second wave). As has been observed in previous studies, mortality rose with age but was consistently lower in the second wave in patients older than 70 years: 35.2% and 27.6% in first and second waves, respectively, which represents a 7.8% absolute risk reduction and 21.6% relative risk reduction.

### Base line characteristics, clinical presentation upon admission, and treatments received between waves

The differences in baseline characteristics (demographics and comorbidities), clinical presentation upon admission, and treatments received between waves were analyzed (Table 1). There were some differences in demographics in second-wave patients, including older age (second wave: 82.0 vs first wave: 80.8 years), a greater proportion of women (second wave: 48.2% vs first wave: 45.2%), a higher proportion of patients with hypertension (second wave: 73.7% vs first wave: 71.3%) and diabetes (second wave: 30.9% vs first wave: 26.3%), and a slightly higher degree of comorbidity (Charlson Comorbidity Index in the second wave: 5.7 vs first wave: 5.4). The clinical manifestations were also slightly different. Laboratory results showed some differences: blood glucose and creatinine values were higher in patients admitted in the second wave whereas hemoglobin was lower. A high-risk inflammatory pattern was more frequent in the first wave. In the second wave, there was greater use of corticosteroids (79.3% vs 39.5%) and remdesivir (12.6% vs 0.4%). Some of these aforementioned variations could be considered protective (e.g., more women, a lower-risk inflammatory pattern) whereas others (higher age, more diabetes, higher creatinine levels) would suggest higher risk of mortality.

**Table 1:** Clinical differences in patients ≥ 70 years admitted in the first and second waves of COVID-19.

Variable	First wave (n = 8587)	Second wave (n = 2386)	OR (95% CI OR)	p
Demographics and comorbidities				
Age (years), mean (SD)	80.8 (7.0)	82.0 (7.1)		< 0.001
Age (years), categorized				< 0.001
70–79 (%)	50.3	41.5	1 (ref.)	
80–89 (%)	38.8	43.8	1.37 (1.24–1.51)	
≥ 90 (%)	10.9	14.8	1.64 (1.43–1.89)	
Female sex (%)	45.2	48.2	1.13 (1.03–1.24)	0.008
Severe dependence (%)^a^	29.1	39.0	1.56 (1.42–1.71)	< 0.001
Age-adjusted Charlson Comorbidity Index, mean (SD)	5.4 (2.1)	5.7 (2.1)		< 0.001
Age-adjusted Charlson Comorbidity Index score, categorized				< 0.001
Moderate (3–4) (%)	39.9	34.2	1 (ref.)	
Severe (> 4) (%)	60.1	65.8	1.27 (1.16–1.40)	
Hypertension (%)	71.3	73.7	1.13 (1.02–1.25)	0.021
Diabetes (%)	26.3	30.9	1.25 (1.13–1.38)	< 0.001
Cardiovascular disease (%)^c^	32.8	32.4	0.98 (0.89–1.08)	0.723
Obesity (%)^b^	20.3	18.7	0.90 (0.80–1.02)	0.095
Obstructive respiratory disease (%)^d^	23.0	22.7	0.99 (0.89–1.10)	0.808
Dementia (%)	18.5	20.9	1.16 (1.04–1.30)	0.009
Malignancy (%)^e^	13.8	14.1	1.03 (0.90–1.17)	0.692
CKD (%)^f^	9.6	10.4	1.10 (0.94–1.27)	0.235
Moderate-severe chronic liver disease (%)	1.1	1.1	1.03 (0.67–1.59)	0.887
Clinical presentation upon admission				
Days from symptom onset, mean (SD)	6.1 (4.7)	5.5 (4.6)		< 0.001
Dyspnea (%)	57.6	58.4	1.03 (0.94–1.13)	0.519
Fatigue (%)	40.4	45.7	1.24 (1.13–1.36)	< 0.001
Anorexia (%)	21.1	23.9	1.18 (1.06–1.31)	0.003
Myalgia (%)	21.8	18.2	0.80 (0.71–0.90)	< 0.001
Clouding of consciousness (%)	20.5	21.1	1.04 (0.93–1.16)	0.486
Diarrhea (%)	19.4	18.3	0.93 (0.83–1.05)	0.241
Abdominal pain (%)	6.3	5.3	0.84 (0.69–1.03)	0.089
Anosmia (%)	4.1	5.1	1.26 (1.02–1.56)	0.035
Sore throat (%)	7.1	5.6	0.78 (0.64–0.95)	0.010
Systolic blood pressure, mmHg, mean (SD)	131.0 (23.1)	131.5 (23.3)		0.418
Arterial stiffness (%)^g^	47.1	48.0	1.04 (0.94–1.13)	0.459
Tachypnea (%)^h^	36.9	32.2	0.81 (0.74–0.89)	< 0.001
Fever (%)^i^	20.5	12.4	0.55 (0.48–0.63)	< 0.001
Tachycardia (%)^j^	16.9	13.7	0.78 (0.68–0.89)	< 0.001
Oxygen saturation (pulse oximetry, %), median [IQR]	93.0 [90.0–96.0]	94.0 [91.0–96.0]		< 0.001
Rales (%)	57.9	51.0	0.76 (0.69–0.83)	< 0.001
pO_2_/FiO_2_ ratio (%), mean (SD)	273.4 (94.3)	290.6 (87.9)		< 0.001
Laboratory and radiological findings at admission				
Hemoglobin (g/dL), mean (SD)	13.2 (2.0)	12.8 (2.1)		< 0.001
Platelet count, ×10^9^/L, median [IQR]	182.0 [141.0–241.0]	185.0 [141.0–247.0]		0.353
White blood cell count, ×10^9^/L, median [IQR]	6.6 [4.9–9.2]	6.6 [4.8–9.4]		0.942
Eosinophil count, ×10^9^/L, median [IQR]	0.0 [0.0–0.0]	0.0 [0.0–0.0]		0.009
Lymphocyte count, ×10^9^/L, median [IQR]	0.9 [0.6–1.2]	0.9 [0.6–1.2]		0.245
Blood glucose (mg/dL), median [IQR]	119.0 [102.0–149.0]	122.0 [104.0–154.0]		< 0.001
Glucose (mg/dL)				< 0.001
< 140 (%)	69.4	65.3	1 (ref.)	
140–179 (%)	16.4	18.6	1.21 (1.07–1.36)	
≥ 180 (%)	14.2	16.1	1.21 (1.06–1.38)	
C-Reactive protein (mg/L), median [IQR]	69.8 [23.2–141.0]	67.6 [27.0–129.8]		0.380
Creatinine (mg/dL), median [IQR]	1.0 [0.8–1.4]	1.0 [0.8–1.5]		0.003
Inflammatory pattern^k^				< 0.001
Low (%)	1.9	1.9	1 (ref.)	
Moderate (%)	13.7	18.0	1.27 (0.88–1.81)	
Severe (%)	84.4	80.1	0.92 (0.65–1.30)	
Infiltrates (any) in chest X-ray (%)	84.5	77.7	0.64 (0.57–0.71)	< 0.001
Treatments received				
Oxygen via high flow nasal cannula (%)	8.1	8.4	1.04 (0.88–1.22)	0.673
Non-invasive mechanical ventilation (%)	5.8	6.4	1.11 (0.92–1.34)	0.269
Invasive mechanical ventilation (%)	5.3	4.5	0.85 (0.69–1.06)	0.142
Systemic corticosteroids (%)	39.5	79.3	5.88 (5.28–6.55)	< 0.001
Remdesivir				< 0.001
No (%)	99.6	87.4	1 (ref.)	
Yes, ≤ 10 days from symptoms onset (%)	0.2	11.5	79.22 (46.20–135.85)	
Yes, > 10 days from symptoms onset (%)	0.2	1.1	5.24 (2.96–9.29)	
Tocilizumab (%)	7.1	7.0	0.98 (0.82–1.17)	0.825
Death (admission or re-admission) (%)	35.2	27.6	0.70 (0.64–0.78)	< 0.001

^a^Severe dependence: Barthel Index < 60; ^b^Obesity: BMI ≥ 30 kg/m2; ^c^Cardiovascular disease: Ischemic heart disease, heart failure, transient ischemic attack, stroke, or peripheral artery disease; ^d^Obstructive respiratory disease: chronic obstructive pulmonary disease, asthma, chronic bronchitis, or obstructive sleep apnea; ^e^Malignancy: solid tumor, leukemia, lymphoma; ^f^CKD: chronic kidney disease (patients on dialysis or with serum creatinine > 3 mg/dL); ^g^Arterial stiffness: pulse pressure ≥ 60 mmHg; ^h^Tachypnea: > 20 breaths per minute; ^i^Fever: temperature > 37.8 °C; ^j^Tachycardia: heart rate > 100 bpm; ^k^Inflammatory pattern: see description in “Methods”. Categoric variables are expressed as percentages and compared using likelihood-ratio chi-square test for statistical significance. OR = odds ratio; 95% CI OR: 95% confidence interval for odds ratio. The odds ratios have been calculated with respect to the first category. Quantitative variables are expressed as mean (standard deviation) or median [interquartile range] and were compared for statistical significance using Student's t-test (with equal or unequal variances) or Mann–Whitney U test, as appropriate.

### Clinical differences in patients ≥ 70 years hospitalized for COVID-19 by survival status

A univariate analysis of mortality was performed. Almost all variables were statistically associated with mortality, denoting the large sample size. Table 2 shows data on demographics, clinical manifestations, laboratory findings, and treatments received. Although some of the associations were strong, the effects were often small.

**Table 2:** Clinical differences in patients ≥ 70 years hospitalized for COVID-19 by survival status.

Variable	Survivors (n = 7290)	No survivors (n = 3683)	OR (95% CI OR)	p
Demographics and comorbidities				
Age (years), mean (SD)	80.0 (6.9)	83.1 (6.9)		< 0.001
Age (years), categorized				< 0.001
70–79 (%)	55.0	35.4	1 (ref.)	
80–89 (%)	35.8	48.0	2.08 (1.91–2.27)	
≥ 90 (%)	9.2	16.7	2.82 (2.49–3.20)	
Female sex (%)	49.0	39.7	0.69 (0.63–0.74)	< 0.001
Severe dependence (%)^a^	25.2	43.5	2.28 (2.10–2.48)	< 0.001
Age-adjusted Charlson Comorbidity Index, mean (SD)	5.2 (2.0)	6.1 (2.2)		< 0.001
Age-adjusted Charlson Comorbidity Index score, categorized				< 0.001
Moderate (3–4) (%)	45.1	25.8	1 (ref.)	
Severe (> 4) (%)	54.9	74.2	2.37 (2.17–2.58)	
Hypertension (%)	70.2	74.9	1.27 (1.16–1.39)	< 0.001
Diabetes (%)	25.9	30.1	1.23 (1.13–1.34)	< 0.001
Cardiovascular disease (%)^c^	28.5	41.0	1.74 (1.60–1.89)	< 0.001
Obesity (%)^b^	20.0	19.8	0.99 (0.89–1.10)	0.836
Obstructive respiratory disease (%)^d^	21.9	24.9	1.18 (1.07–1.29)	< 0.001
Dementia (%)	15.4	26.2	1.94 (1.76–2.14)	< 0.001
Malignancy (%)^e^	12.6	16.2	1.34 (1.19–1.49)	< 0.001
CKD (%)^f^	7.7	13.9	1.93 (1.70–2.19)	< 0.001
Moderate-severe chronic liver disease (%)	1.1	1.1	1.05 (0.72–1.53)	0.788
Clinical presentation upon admission				
Days from symptom onset, mean (SD)	6.4 (4.8)	5.1 (4.3)		< 0.001
Dyspnea (%)	51.2	70.9	2.32 (2.13–2.53)	< 0.001
Fatigue (%)	42.7	39.2	0.87 (0.80–0.94)	< 0.001
Anorexia (%)	21.4	22.4	1.07 (0.97–1.17)	0.202
Myalgia (%)	23.2	16.5	0.65 (0.59–0.72)	< 0.001
Clouding of consciousness (%)	14.2	33.4	3.02 (2.74–3.32)	< 0.001
Diarrhea (%)	21.6	14.4	0.61 (0.55–0.68)	< 0.001
Abdominal pain (%)	6.7	4.7	0.69 (0.58–0.83)	< 0.001
Anosmia (%)	5.8	1.3	0.22 (0.16–0.30)	< 0.001
Sore throat (%)	7.6	5.1	0.66 (0.55–0.78)	< 0.001
Systolic blood pressure, mmHg, mean (SD)	132.5 (22.4)	128.5 (24.2)		< 0.001
Arterial stiffness (%)^g^	48.4	45.2	0.88 (0.81–0.95)	0.002
Tachypnea (%)^h^	26.3	55.0	3.44 (3.16–3.74)	< 0.001
Fever (%)^i^	17.0	22.2	1.39 (1.26–1.54)	< 0.001
Tachycardia (%)^j^	13.4	21.7	1.79 (1.62–1.99)	< 0.001
Oxygen saturation (pulse oximetry, %), median [IQR]	94.0 [91.0–96.0]	91.0 [86.0–95.0]		< 0.001
Rales (%)	53.6	61.9	1.41 (1.30–1.53)	< 0.001
pO_2_/FiO_2_ ratio (%), mean (SD)	298.9 (85.7)	239.3 (93.5)		< 0.001
Laboratory and radiological findings at admission				
Hemoglobin (g/dL), mean (SD)	13.2 (1.9)	13.0 (2.2)		< 0.001
Platelet count, × 10^9^/L, median [IQR]	185.0 [143.0–246.5]	178.0 [138.0–233.0]		< 0.001
White blood cell count, × 10^9^/L, median [IQR]	6.3 [4.8–8.6]	7.4 [5.3–10.6]		< 0.001
Eosinophil count, × 10^9^/L, median [IQR]	0.0 [0.0–0.0]	0.0 [0.0–0.0]		< 0.001
Lymphocyte count, × 10^9^/L, median [IQR]	0.9 [0.7–1.3]	0.8 [0.5–1.1]		< 0.001
Blood glucose (mg/dL), median [IQR]	116.0 [100.0–142.0]	129.0 [108.0–169.0]		< 0.001
Glucose (mg/dL)				< 0.001
< 140 (%)	73.2	59.2	1 (ref.)	
140–179 (%)	15.3	19.9	1.60 (1.44–1.79)	
≥ 180 (%)	11.4	20.9	2.26 (2.02–2.53)	
C-Reactive protein (mg/L), median [IQR]	56.5 [19.0–116.9]	101.0 [40.9–179.0]		< 0.001
Creatinine (mg/dL), median [IQR]	0.9 [0.8–1.2]	1.2 [0.9–1.7]		< 0.001
Inflammatory pattern^k^				< 0.001
Low (%)	2.6	0.4	1 (ref.)	
Moderate (%)	19.1	6.1	1.88 (1.09–3.26)	
Severe (%)	78.3	93.4	7.01 (4.13–11.90)	
Infiltrates (any) in chest X-ray (%)	80.8	87.4	1.65 (1.47–1.85)	< 0.001
Treatments received				
Oxygen via high flow nasal cannula (%)	5.3	13.7	2.82 (2.45–3.24)	< 0.001
Non-invasive mechanical ventilation (%)	3.0	11.7	4.24 (3.59–5.02)	< 0.001
Invasive mechanical ventilation (%)	2.9	9.5	3.49 (2.93–4.16)	< 0.001
Systemic corticosteroids (%)	45.1	54.4	1.46 (1.35–1.58)	< 0.001
Remdesivir				< 0.001
No (%)	96.4	98.0	1 (ref.)	
Yes, ≤ 10 days from symptoms onset (%)	3.1	1.7	0.53 (0.40–0.71)	
Yes, > 10 days from symptoms onset (%)	0.5	0.3	0.65 (0.34–1.26)	
Tocilizumab (%)	6.6	8.1	1.24 (1.07–1.45)	0.005

^a^Severe dependence: Barthel Index < 60; ^b^Obesity: BMI ≥ 30 kg/m^2^; ^c^Cardiovascular disease: Ischemic heart disease, heart failure, transient ischemic attack, stroke, or peripheral artery disease; ^d^Obstructive respiratory disease: chronic obstructive pulmonary disease, asthma, chronic bronchitis, or obstructive sleep apnea; ^e^Malignancy: solid tumor, leukemia, lymphoma; ^f^CKD: chronic kidney disease (patients on dialysis or with serum creatinine > 3 mg/dL); ^g^Arterial stiffness: pulse pressure ≥ 60 mmHg; ^h^Tachypnea: > 20 breaths per minute; ^i^Fever: temperature > 37.8 ºC; ^j^Tachycardia: heart rate > 100 bpm; ^k^Inflammatory pattern: see description in “Methods”. Categoric variables are expressed as percentages and compared using likelihood-ratio chi-square test for statistical significance. OR = odds ratio; 95% CI OR: 95% confidence interval for odds ratio. The odds ratios have been calculated with respect to the first category. Quantitative variables are expressed as mean (standard deviation) or median [interquartile range] and were compared for statistical significance using Student's t-test (with equal or unequal variances) or Mann–Whitney U test, as appropriate.

The variables and interactions included in the maximal logistic regression model estimated are shown in Table 3.

**Table 3:** Logistic regression multivariate model showing the average effect of being admitted in the second wave after adjusting for confounding variables.

Death	Adjusted odds ratio (95% CI)	p-value
Second wave	0.35 (0.24–0.52)	< 0.001
Age (years)		
70–79	1 (ref.)	
80–89	1.88 (1.65–2.15)	< 0.001
≥ 90	2.32 (1.91–2.81)	< 0.001
Female sex	0.64 (0.57–0.71)	< 0.001
Age-adjusted Charlson comorbidity index		
Moderate (3–4)	1 (ref.)	
Severe (> 4)	1.45 (1.27–1.66)	< 0.001
Arterial hypertension	1.00 (0.89–1.14)	0.957
Arterial stiffness^a^	0.86 (0.77–0.96)	0.007
Severe dependence^b^	1.65 (1.42–1.91)	< 0.001
Days from beginning of symptoms	0.95 (0.94–0.96)	< 0.001
Clouding of consciousness	1.76 (1.54–2.02)	< 0.001
Tachypnea	1.92 (1.71–2.15)	< 0.001
Oxygen saturation/FiO_2_ ratio	1.00 (0.99–1.00)	< 0.001
Blood glucose (mg/dL)		
< 140	1 (ref.)	
140–179	1.25 (1.08–1.44)	0.003
≥ 180	1.45 (1.24–1.68)	< 0.001
Inflammatory pattern^c^		
Low	1 (ref)	
Moderate	1.44 (0.75–2.78)	0.277
Severe	3.66 (1.94–6.92)	< 0.001
Bilateral pneumonia	1.37 (1.22–1.55)	< 0.001
Tocilizumab	0.91 (0.73–1.12)	0.372
Remdesivir, start date		
No	1 (ref.)	
Yes, ≤ 10 days from beginning of symptoms	0.52 (0.36–0.75)	0.001
Yes, > 10 days from beginning of symptoms	0.63 (0.26–1.50)	0.294
Systemic corticosteroids	1.14 (1.00–1.29)	0.047
Ventilatory support		
No	1 (ref.)	
Non-invasive	4.09 (3.41–4.89)	< 0.001
Invasive	6.36 (4.99–8.11)	< 0.001
Wave#severe dependence^d^	1.50 (1.14–1.97)	0.004
Wave#systemic corticosteroids^e^	1.59 (1.09–2.33)	0.017
Constant	0.31 (0.15–0.64)	0.002

^a,c^See description in “Methods”. ^b^Barthel index < 60. ^d^Interaction between wave and severe dependence. ^e^Interaction between wave ant systemic corticosteroid therapy. The odds ratio of Second wave represents the average effect of hospital admission during the second wave, adjusted for confounding terms and interaction. Table 4 displays the effect for each combination of interaction term values.

The odds ratios and risk ratios of mortality of being admitted in the second wave versus the first one for the four different combinations of interaction covariates values are shown in Table 4. The protective effect of being admitted during the second wave of COVID-19 is highest for patients without severe dependence who are not treated with corticosteroids, and it diminishes when either of these circumstances is present, becoming neutral when both converge.

**Table 4:** Effect of second wave on mortality in patients ≥ 70 years hospitalized for COVID-19.

Severe dependence (Barthel index < 60)	Treatment with systemic corticosteroids	AOR (95% CI AOR)	ARR (95% CI ARR)
No	No	0.35 (0.24–0.52)	0.54 (0.42–0.69)
No	Yes	0.56 (0.45–0.69)	0.73 (0.65–0.82)
Yes	No	0.52 (0.37–0.75)	0.73 (0.60–0.88)
Yes	Yes	0.83 (0.67–1.04)	0.92 (0.84–1.02)

AOR: adjusted odds ratio; ARR: adjusted risk ratio calculated with delta-method standard errors for the wave covariate.

## Discussion

This study confirms a difference in mortality in patients hospitalized for COVID-19 in Spain between the first and the second wave of the pandemic. The lower mortality rate observed in the second wave is due to the lower mortality in patients ≥ 70 years; no differences in mortality were observed among younger subjects. To our knowledge, this is a novel finding that has not been previously described.

This reduced mortality rate found in older patients hospitalized during the second wave of the COVID-19 pandemic compared to the first wave could be due to some unmeasured or unknown confounders which may be broadly grouped into three categories: differences in the patients, differences in the disease, or differences in treatment and overall management.

The second-wave patients in this study were slightly older, had a higher degree of dependence (as measured by Barthel index), and a greater comorbidity burden. Given that these factors are associated with a worse COVID-19 prognosis, the clinical and epidemiologic differences between patients hospitalized in the first and the second wave^20^ do not explain the lower mortality observed in the second wave.

Although patients admitted during the second wave were slightly less severe at admission, after adjustments in the multivariate analysis, admission during the second wave remained an independent protective factor. In the second wave, the use of treatments that have been shown to reduce COVID-19 mortality increased, namely corticosteroids^21^, tocilizumab^22,23^, and remdesivir^24^. However, the lower mortality in the second wave was unchanged after adjusting for the use of these therapies.

**Figure 2:**
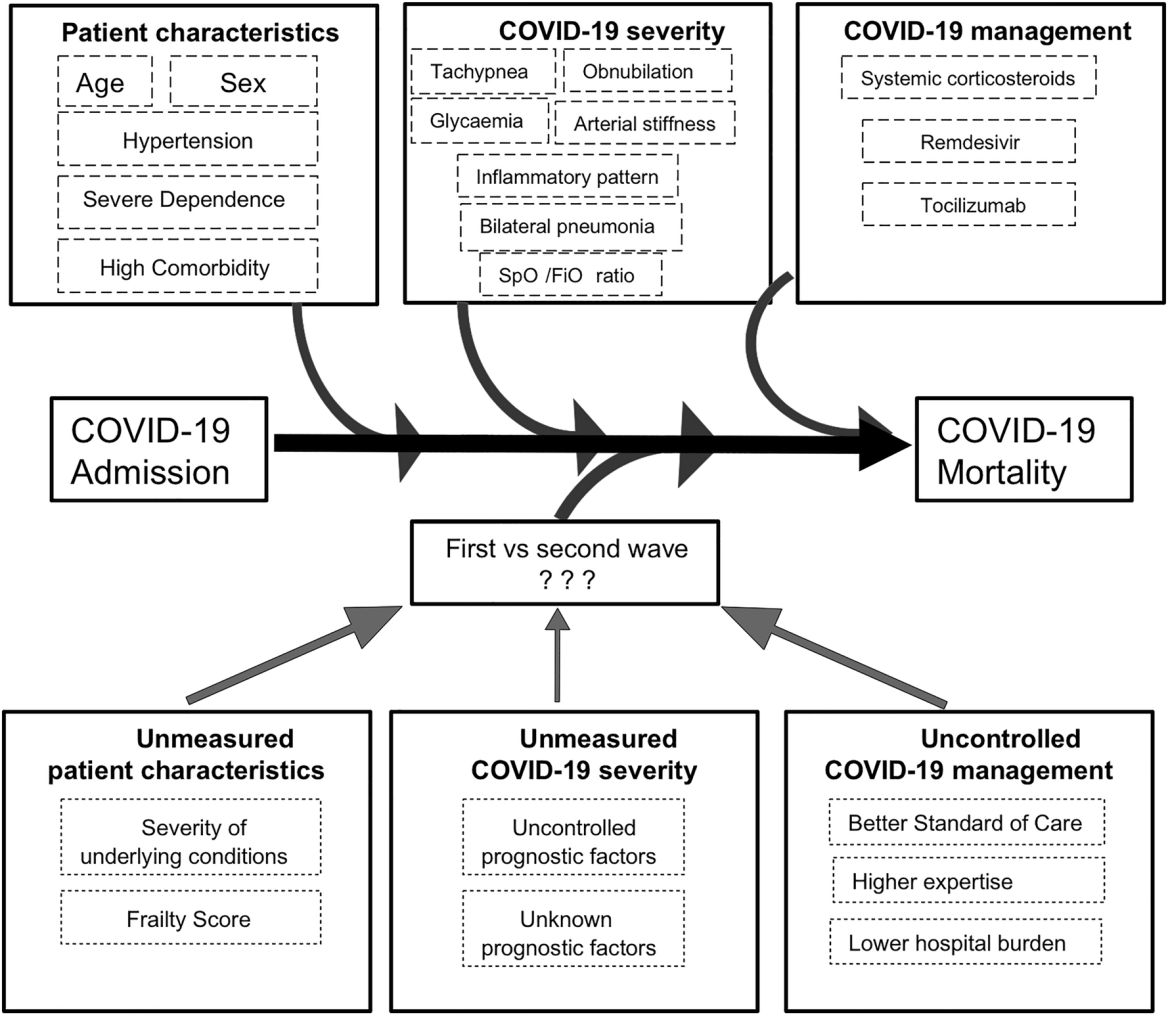
Several characteristics of patients, differences in COVID-19 severity and management could confound the estimation of mortality between waves. After adjusting for these factors, differences in the outcome could be explained by unmeasured patient characteristics and COVID-19 severity factors, or uncontrolled COVID-19 management.

Our results suggest that there may be some factor (or, more probably, combination of factors), associated with hospitalization that influences mortality and changed between the waves. Some potential candidates are changes in the overall management of patients, improvements in clinical expertise, and a lesser degree of hospital overload (Fig. 2).

Interestingly, the analysis of the interactions between severe dependency and systemic corticosteroid treatment with the hospital admission wave has allowed us to discover that the protective effect of the second wave on mortality is highest when both factors are absent, somewhat reduced when either of them is present, and neutral when both are present. It is likely, then, that the factors, largely unknown, that explain an average protective effect of the second wave, may not succeed in improving the prognosis of the most fragile and severe patients, such as those requiring corticosteroid treatment.

We have learned that there are a lot of “intangibles” that influence the prognosis of COVID-19 hospitalization. Quick identification of respiratory failure, thromboprophylaxis and early mobilization, an adequate state of hydration, proper management of stress hyperglycemia, nutritional support, physical rehabilitation, and more have become the new standard of care and are potential uncontrolled factors that could explain the better prognosis in the second wave. Most of these factors will have a greater impact on the older people, as they are frailer and thus prone to physical deconditioning, dehydration, or confusional states.

Another possibility is that greater clinical expertise led to improved prognosis. Indeed, in the USA, a progressive decline in COVID-19-related mortality was described after the passage of just a few months^9^.

Healthcare system overload could be an important driver of mortality in the COVID-19 pandemic^25–27^. The first wave in Spain was explosive and overwhelmed hospitals in some areas. For instance, in Madrid, COVID-19 occupancy reached nearly 300% of the nominal ICU capacity and nearly 105% of the general ward capacity^28^, paralyzing non-emergency surgical procedures. On the contrary, the second wave has been less dramatic, leading to a smaller impact on hospital occupancy and healthcare activity. It may well be that the lower mortality in the second wave is mainly a reflection of less healthcare system overload. As our study does not include data on the true workload borne by the hospitals, this notion remains a hypothesis.

In extreme cases, healthcare system overload leads to shortages, which can also have a greater effect on the older persons due to implementation of triage criteria. If it were confirmed that healthcare system overload causes greater mortality in the older persons, it would be a moral imperative for us as a society to quickly adopt robust preventative measures as soon as another wave is upon us and there is risk of healthcare system overload. Our registry cannot answer this crucial question, as we lack data on hospital or ICU patient loads at the time of the patients’ admissions.

We recognize several limitations in our study. The large number of researchers involved and/or variability in the availability of data from each hospital could have led to information bias. Selection bias could have been introduced given the voluntary participation of each center. A potential source of uncontrolled confounding factors is the severity of underlying conditions and overall frailty of patients. The lower mortality in the second wave could also be explained by a “harvesting effect” that may be present if the most severely ill patients had already died in the first wave, though patients admitted during the second wave were older and had more dementia and comorbidities. However, we did not analyze frailty, a prognostic factor that is more potent than dependence or age in older patients^20^.

In terms of limitations regarding treatment-related variables, the effect of remdesivir and tocilizumab on mortality are strongly time-dependent with a narrow window of opportunity and both tocilizumab and corticosteroids are indicated for a worsening respiratory or inflammatory condition. Our registry includes data on the timing of the drug initiation but does not include clinical and laboratory findings at that moment, so it is not possible to evaluate the exact effect of the drugs. The deleterious effect of corticosteroids or tocilizumab in our multivariate analysis should be interpreted as a marker of the patient’s worsening condition.

The strengths of this study include its multicenter, nationwide design as well as the large number of patients included, which provides strong statistical power. The consecutive inclusion of patients in each center limits selection bias.

In conclusion, mortality in the older patients hospitalized in Spain with COVID-19 has been significantly lower in the second wave even after adjusting for baseline clinical condition, disease severity upon admission, and pharmacological treatment with proven benefits in treating COVID-19, except in severely dependent persons in need of corticosteroid treatment. Our results suggest that this reduction of mortality could be related to a better standard of care, improvements in clinical expertise, less healthcare system overload, or a combination of these three factors, though other unknown confounding factors cannot be ruled out.

## Methods

### Study design

This is a retrospective cohort study comparing the first and second waves of the COVID-19 epidemic in Spain. The first wave was defined as the period between January 1 and July 7, 2020. The second wave was defined as the period between July 8, 2020 and December 26, 2020, before SARS-CoV-2 vaccination was available.

The final weeks of the first wave and the initial weeks of the second one thus defined periods with a low incidence of COVID-19 and few hospital admissions. However, this cut-off point reflects the transition from the greater healthcare system overload which occurred in the initial months to the lesser healthcare system overload of the later months.

### Registry design

The SEMI-COVID-19 Registry is an ongoing, nationwide, retrospective cohort launched in March 2020 that comprises most consecutive patients hospitalized in Spain who are discharged with confirmed COVID-19 disease. It has become one of the largest repositories of COVID-19 patient data and includes more than 20,000 patients to date. Its characteristics have been thoroughly described elsewhere^6^.

Inclusion criteria for the registry were age ≥ 18 years and first hospital discharge with a confirmed diagnosis of COVID-19. Exclusion criteria were subsequent admissions of the same patient and denial or withdrawal of informed consent.

Consecutive patients who required hospital admission and who had SARS-CoV-2 infection confirmed by a positive result on real-time polymerase chain reaction (RT-PCR) testing of a nasopharyngeal, bronchoalveolar lavage, or sputum sample and who provided verbal consent were included in the registry. With the advent of the second wave, the inclusion criteria were expanded with two modifications: antigen testing was accepted as a method for confirming diagnosis and reinfections (> 3 months from the initial infection) of the same patient were accepted for inclusion. From March 23, 2020, to December 26, 2021, a total of 22,494 patients from 126 hospitals throughout the country were included in the registry.

Patients were treated at their attending physician’s discretion according to local protocols and clinical judgment. Patients included in open-label clinical trials could be included in the registry provided that all information about treatment was available. Due to its observational nature, the registry caused no inconvenience to the patients included.

### Data collection

Clinical investigators all over the country collected data from medical records using a standardized online data capture system (DCS). The DCS includes both a database manager and the set of procedures for the verification of data. Patient identifiable data are dissociated and pseudonymized using an alphanumeric sequence and each researcher keeps a protected registry (patient log) for the purpose of data verification and quality control. The database platform is hosted in a secure server and both the database and each client–server transfer are encrypted. The pseudonymization system allows for safeguarding patient privacy while also complying with ethical considerations and data protection regulations.

Data on more than 300 variables are collected retrospectively after patient discharge and grouped under various headings: inclusion criteria, epidemiological data, RT-PCR and serology data, prior comorbidities and medication history, findings (symptoms and physical examination) at admission, laboratory (blood gases, metabolic panel, complete blood count, coagulation) and diagnostic imaging tests at admission, additional data at seven days after admission or at admission to the intensive care unit, pharmacological treatment (antiviral drugs, immunomodulators, antibiotics) and ventilatory support during the hospitalization, complications during the hospitalization, and progress after discharge and/or 30 days from diagnosis. The variables in the registry have previously been described^6^.

A number of secondary variables were calculated from the primary variables in the registry. Some qualitative variables were classified into binary categories whereas some quantitative variables were categorized as normal or abnormal; age was categorized into decades. Arterial stiffness was defined as a pulse pressure greater than or equal to 60 mmHg^29^. Blood glucose levels were categorized into three groups according to standard glycemic targets in hospitalized patients: < 140 mg/dl, 140–180 mg/dl, and > 180 mg/dl^30^. The risk categories based on the pattern of inflammation used in this study were a modified version of risk categories recently reported in another work from the SEMI-COVID-19 Registry^31^. The low-risk category was defined as lactate dehydrogenase (LDH), C-reactive protein (CRP), and D-dimer (DD) values in the first tercile and lymphocyte count in the third tercile. The high-risk category was defined as any LDH, CRP, or DD values in the third tercile or lymphocyte count in the first tercile. The moderate-risk category was defined as patients who did not meet the criteria of the low- or high-risk categories.

### Statistical analysis

In a descriptive analysis, we compared epidemiological data, demographics, signs and symptoms on admission, comorbidities, laboratory results, chest x-ray findings, treatment received, and clinical outcomes. Continuous variables were expressed as mean and standard deviation or median and interquartile range (IQR), according to distribution assessed by the Shapiro–Wilk test and standardized normal probability plots. Categorical variables were expressed as frequencies and percentages. Differences between groups were compared using Student’s t-test or the Mann–Whitney U test for continuous variables and the likelihood-ratio chi-square test for categorical variables.

A univariate analysis was performed to explore possible risk factors for all-cause death during admission or the next 30 days from discharge and variables associated with the exposure (pandemic wave) using binomial logistic regressions. The variables were chosen from an array of clinical and laboratory findings, previous comorbidities, and treatments received according to local protocols. Due to the large sample size, almost all variables showed significant differences in the comparisons between exposure and outcome groups in the univariate analysis.

We created a logistic regression model to assess the effect of being admitted during the first or second wave on all-cause mortality risk. We selected a series of predictors associated with the exposure (pandemic wave) and the outcome (mortality) as potential confounding factors. The selection criteria also took theoretical arguments or findings from other studies into consideration in order to adjust for factors that could explain a potential difference in the risk of death between the two waves.

The admitting variables that were ultimately included as possible confounders of the wave effect were age (categorized into decades from 70 years), sex (reference: male), age-adjusted Charlson Comorbidity Index (reference: moderate comorbidity), degree of dependence (reference: none or mild dependence), hypertension, arterial stiffness (pulse pressure ≥ 60 mmHg), clouding of consciousness, tachypnea, oxygen saturation/FiO2 ratio (%), blood glucose level categories, risk category based on the pattern of inflammation, and bilateral pneumonia as well as tocilizumab, remdesivir, or corticosteroid therapy during hospitalization.

In addition, first-order interactions between the waves and all potential confounding factors were included in the initial model. Multicollinearity was detected for several terms of interaction, and they were removed from the model. A chunk test for the rest of interaction terms did show statistical significance (p < 0.001), so individual likelihood ratio tests were performed for every one of them. Three interactions with the variable "Wave" remained statistically significant: severe dependency, corticosteroid treatment, and ventilatory support, which has three different categories. In order to achieve an interpretable estimation and reduce the number of combinations for which to calculate the wave effect on mortality, we decided to omit the interaction of the wave with ventilatory support. So, the final logistic regression model included all the confusion terms and the interactions of Wave with Severe dependence and Systemic corticosteroid therapy. We did not conduct variable selection once the model was estimated, as this maximal model is the best fit for calculating the wave's effect on mortality. Adjusted odds ratios and risk ratios were estimated for each combination of the values of interaction terms. Adjusted risk ratios were calculated with delta-method standard errors for the wave covariate. All analyses were conducted using Stata version 18.0 (StataCorp. 2023. Stata Statistical Software: Release 18. College Station, TX: StataCorp LLC).

### Ethical considerations

The SEMI-COVID-19 Registry was approved by the Provincial Research Ethics Committee of Málaga (Spain) on March 27, 2020 (Ethics Committee code: SEMI-COVID-19 27/03/20). All experimental protocols were approved by Ethic Committee of Infanta Cristina University Hospital, Ethic Committee of Gregorio Marañón University Hospital, Ethic Committee of Costa del Sol Hospital. Marbella, Cabueñes University Hospital, Ethic Committee of Complejo Hospitalario Universitario de Albacete, Ethic Committee of Hospital Universitario La Paz, Ethic Committee of Hospital Royo Villanova, Ethic Committee of Complejo Hospitalario Universitario de Santiago, Ethic Committee of Hospital Universitario Puerta de Hierro, Ethic Committee of Hospital Universitario Doctor Peset, Ethic Committee of Hospital Clínico San Carlos, Ethic Committee of Complejo Asistencial de Segovia, Ethic Committee of Complejo Hospital Universitario de Badajoz, Ethic Committee of Hospital Universitario Miguel Servet, Ethic Committee of Hospital Universitario de la Princesa, Ethic Committee of Hospital Universitario Infanta Sofía, Ethic Committee of Complexo Hospitalario Universitario A Coruña, Ethic Committee of Hospital de Sant Joan Despí Moisès Broggi, Hospital Universitari Sant Joan d'Alacant, and Ethic Committee of 12 de Octubre University Hospital. The processing of personal data strictly complied with Spanish Law 14/2007, of July 3, on Biomedical Research; Regulation (EU) 2016/679 of the European Parliament, and of the Council of April 27, 2016, on the protection of natural persons with regard to the processing of personal data and on the free movement of such data, and repealing Directive 95/46/EC (General Data Protection Regulation); and Spanish Organic Law 3/2018, of December 5, on the Protection of Personal Data and the Guarantee of Digital Rights. Informed consent, written or verbal, was obtained from all participants. In the periods of maximum hospital care pressure with high number of cases admitted, a written informed consent was not possible to obtain if overwork left no time to explain informed consent, prepared the written documentation and keep safe it for overwork (March to April 2020, November to December 2020). In these cases, it was noted noted on the medical record that a written informed consent was not possible to obtain, but the patient gave verbal consent, as such procedure was approved by the ethics committees.

All methods were carried out in accordance with relevant guidelines and regulations. The STROBE Statement guidelines^32^ were followed in the conduct and reporting of the study.

## Data Availability

The datasets used and/or analyzed during the current study are available from the corresponding author on reasonable request.

## References

[1] Weekly epidemiological update on COVID-19-15 June 2021. https://www.who.int/publications/m/item/weekly-epidemiological-update-on-covid-19---15-june-2021 (Accessed 20 June 2021).

[2] Zhou F, Yu T, Du R (2020). Clinical course and risk factors for mortality of adult inpatients with COVID-19 in Wuhan, China: A retrospective cohort study. Lancet.

[3] Docherty AB, Harrison EM, Green CA (2020). Features of 20 133 UK patients in hospital with covid-19 using the ISARIC WHO Clinical Characterisation Protocol: Prospective observational cohort study. BMJ.

[4] Richardson S, Hirsch JS, Narasimhan M (2020). Presenting characteristics, comorbidities, and outcomes among 5700 patients hospitalized with COVID-19 in the New York City area. JAMA.

[5] Borobia AM, Carcas AJ, Arnalich F (2020). A cohort of patients with COVID-19 in a major teaching hospital in Europe. J. Clin. Med..

[6] Casas-Rojo JM, Antón-Santos JM, Millán-Núñez-Cortés J (2020). Clinical characteristics of patients hospitalized with COVID-19 in Spain: Results from the SEMI-COVID-19 Registry. Rev. Clin. Esp..

[7] Ramos-Rincon J-M, Buonaiuto V, Ricci M (2020). Clinical characteristics and risk factors for mortality in very old patients hospitalized with COVID-19 in Spain. J. Gerontol. A Biol. Sci. Med. Sci..

[8] Finelli L, Gupta V, Petigara T, Yu K, Bauer KA, Puzniak LA (2021). Mortality among US patients hospitalized with SARS-CoV-2 infection in 2020. JAMA Netw. Open..

[9] Asch DA, Sheils NE, Islam MN (2021). Variation in US hospital mortality rates for patients admitted with COVID-19 during the first 6 months of the pandemic. JAMA Intern. Med..

[10] Vahidy FS, Drews AL, Masud FN (2020). Characteristics and outcomes of COVID-19 patients during initial peak and resurgence in the Houston metropolitan area. JAMA.

[11] Docherty AB, Mulholland RH, Lone NI (2021). Changes in in-hospital mortality in the first wave of COVID-19: A multicentre prospective observational cohort study using the WHO Clinical Characterisation Protocol UK. Lancet Respir. Med..

[12] Fan G, Yang Z, Lin Q, Zhao S, Yang L, He D (2020). Decreased Case fatality rate of COVID-19 in the second wave: A study in 53 countries or regions. Transbound Emerg. Dis..

[13] James N, Menzies M, Radchenko P (2021). COVID-19 second wave mortality in Europe and the United States. Chaos.

[14] Nørgaard SK, Vestergaard LS, Nielsen J (2021). Real-time monitoring shows substantial excess all-cause mortality during second wave of COVID-19 in Europe, October to December 2020. Euro Surveill..

[15] Radovanovic D, Pini S, Franceschi E (2021). Characteristics and outcomes in hospitalized COVID-19 patients during the first 28 days of the spring and autumn pandemic waves in Milan: An observational prospective study. Respir Med..

[16] Borghesi A, Golemi S, Carapella N, Zigliani A, Farina D, Maroldi R (2021). Lombardy, Northern Italy: COVID-19 second wave less severe and deadly than the first? A preliminary investigation. Infect. Dis..

[17] Salyer SJ, Maeda J, Sembuche S (2021). The first and second waves of the COVID-19 pandemic in Africa: A cross-sectional study. Lancet.

[18] de Souza FSH, Hojo-Souza NS, da Silva CM, Guidoni DL (2021). Second wave of COVID-19 in Brazil: Younger at higher risk. Eur. J. Epidemiol..

[19] Iftimie S, López-Azcona AF, Vallverdú I (2021). First and second waves of coronavirus disease-19: A comparative study in hospitalized patients in Reus, Spain. PLoS ONE.

[20] Hewitt J, Carter B, Vilches-Moraga A (2020). The effect of frailty on survival in patients with COVID-19 (COPE): A multicentre, European, observational cohort study. Lancet Public Health..

[21] Horby P, Lim WS, RECOVERY Collaborative Group (2021). Dexamethasone in hospitalized patients with Covid-19. N. Engl. J. Med..

[22] Gordon AC, Mouncey PR, REMAP-CAP Investigators (2021). Interleukin-6 receptor antagonists in critically ill patients with Covid-19. N. Engl. J. Med..

[23] RECOVERY Collaborative Group (2021). Tocilizumab in patients admitted to hospital with COVID-19 (RECOVERY): A randomised, controlled, open-label, platform trial. Lancet.

[24] Beigel JH, Tomashek KM, Dodd LE (2020). Remdesivir for the treatment of Covid-19—final report. N. Engl. J. Med..

[25] Soria A, Galimberti S, Lapadula G (2021). The high volume of patients admitted during the SARS-CoV-2 pandemic has an independent harmful impact on in-hospital mortality from COVID-19. PLoS ONE.

[26] Ji Y, Ma Z, Peppelenbosch MP, Pan Q (2020). Potential association between COVID-19 mortality and health-care resource availability. Lancet Glob. Health..

[27] African COVID-19 Critical Care Outcomes Study (ACCCOS) Investigators (2021). Patient care and clinical outcomes for patients with COVID-19 infection admitted to African high-care or intensive care units (ACCCOS): A multicentre, prospective, observational cohort study. Lancet.

[28] Condes E, Arribas JR (2021). Impact of COVID-19 on Madrid hospital system. Enferm Infecc. Microbiol. Clin..

[29] Rodilla E, Lopez-Carmona MD, Cortes X (2020). Impact of arterial stiffness on all-cause mortality in patients hospitalized with COVID-19 in Spain. Hypertension.

[30] Carrasco-Sánchez FJ, López-Carmona MD, Martínez-Marcos FJ (2021). Admission hyperglycaemia as a predictor of mortality in patients hospitalized with COVID-19 regardless of diabetes status: Data from the Spanish SEMI-COVID-19 Registry. Ann. Med..

[31] Rubio-Rivas M, Corbella X, Formiga F (2021). Risk categories in COVID-19 based on degrees of inflammation: Data on More than 17,000 patients from the Spanish SEMI-COVID-19 Registry. J. Clin. Med..

[32] von Elm E, Altman DG, Egger M (2007). The Strengthening the Reporting of Observational Studies in Epidemiology (STROBE) statement: Guidelines for reporting observational studies. Lancet.

